# Barriers to diabetic foot care in a developing country with a high incidence of diabetes related amputations: an exploratory qualitative interview study

**DOI:** 10.1186/s12913-015-1043-5

**Published:** 2015-09-14

**Authors:** Cornelia Guell, Nigel Unwin

**Affiliations:** Public Health Group, Faculty of Medical Sciences, University of the West Indies, Cave Hill Campus, Bridgetown, Barbados; MRC Epidemiology Unit and UKCRC Centre for Diet and Activity Research (CEDAR), University of Cambridge, Cambridge, UK; Chronic Disease Research Centre, University of the West Indies, Bridgetown, Barbados

## Abstract

**Background:**

Diabetes related foot disease is a major cause of morbidity and mortality in people with diabetes. This is despite the fact that interventions to reduce the burden of diabetic foot disease are estimated to be highly cost effective, even cost saving in both developed and developing countries. This exploratory qualitative study was undertaken in a developing country known to have a very high rate of diabetes related amputations. The aim of the study was to explore barriers to foot care from the perspectives of health care professionals and patients, with a view to informing further work to develop effective interventions.

**Methods:**

Semi-structured interviews, each of 30 to 60 minutes, were conducted with a purposive sample of 20 individuals (11 health carers and 9 patients with diabetes). Participants were asked how diabetic foot care was experienced and practised, and about knowledge and attitudes relevant to care. Health carers were also asked how they negotiated issues of priority setting within the available resources. Interviews were recorded, transcribed and underwent thematic analysis.

**Results:**

Three broad categories of potential barriers to diabetic foot care were identified. First, health carers reported that they and their patients tended to *prioritise glycaemic control* and that this often eclipsed foot care. Second, health carers described *resistance to changing professional roles,* particularly within the context of limited resources*.* Newly assigned foot screening and care duties did not fit in easily with their main work schedule. The overall effect of this was to lead to increased referrals to already overstretched, and difficult to access, podiatrists. Finally, patients reported a health care system with significant *reliance on ‘self-care’ ability,* including the need for time and expertise to negotiate access to scarce professional foot care appointments.

**Conclusions:**

The findings from this exploratory study provide insight on broad barriers to diabetic foot care within a developing country setting. The three areas identified deserve further investigation to determine their impact on the delivery of diabetic foot care and the implications for designing effective interventions.

## Background

The vast majority of people with diabetes, around 80 %, live in ‘developing’ countries, and it is in these countries that the largest increases in the burden of diabetes will occur over the coming decades [1]. Diabetic foot problems are a major cause of morbidity and premature mortality in people with diabetes, and contribute substantially to the health care costs associated with diabetes [2–4]. Interventions to reduce the burden of diabetic foot ulceration and amputation are estimated to be highly cost-effective, indeed cost saving, in both developed and developing country settings [5, 6]. The challenge, particularly in less well-resourced health care systems, is how to implement effective foot care that realises these potential health gains and cost savings.

We undertook this exploratory qualitative study in a setting reported to have one of the highest rates of diabetes related amputations in the world [7]. Our aim was to better understand from the perspectives of people with diabetes and their health care providers the broad types of barriers they faced achieving effective foot care. Our overall goal was to inform further work towards the development of interventions. We were particularly interested in how foot care is prioritised by health care providers within a resource limited health system and by patients within the competing demands of their everyday lives. This study complements previously published qualitative work on the diabetic foot, which has tended to focus on personal beliefs and behaviours and has been largely from developed country settings [8–10].

## Methods

### Setting

The study was undertaken in the Caribbean nation of Barbados, a small island developing state [11] with a total population of around 280,000 [12]. Overall rates of diabetes in the Caribbean are the highest in the Americas, and some of the highest in the world [1]. In Barbados 14.6 % of the population aged 20 to 79 is estimated to have diabetes, just under 30,000 people [1]. The reported incidence of diabetes related lower extremity amputations in Barbados is one of the highest in the world [7] with mortality following amputation exceeding 50 % at 5 years [13].

Diabetes care in Barbados is provided by both public and private health care systems. The public healthcare system is largely free at the point of access for all citizens of Barbados. Public primary care is provided across eight polyclinics, and there is one public hospital on the island. At the time of the study there were three podiatrists serving the public health care system. In response to the high incidence of amputations and limited specialist foot care resources the Ministry of Health of supported the introduction of the International Diabetes Federation Step-by-Step foot care programme in 2009, a ‘train the trainer’ programme that aims to devolve foot care to polyclinic nurses and doctors [14]. Polyclinic nurses and doctors received training in several steps, first learning to examine and identify diabetic foot disease in a basic course (in 2009), then learning in an advanced course how to treat basic foot problems such as corn or hard skin (in 2010). Alongside the educational programme, forms were introduced to document and monitor foot examinations. Participants of Step-by-Step also learn how to train their colleagues. No formal evaluation of this programme has been undertaken to document either coverage or potential impact, although the strong impression from health care workers is that both have been limited.

### Study participants

Between May and August 2012, 20 semi-structured interviews were conducted with nine patients of two Barbadian public polyclinics, and 10 health care professionals (four doctors, four nurses and two podiatrists) working in these polyclinics. The two polyclinics were purposively chosen, with the help of the NCD focal point at the Ministry of Health, to reflect areas of different socio-economic status. One polyclinic was in a relatively well off, or ‘middle-class’, rural area, and the other was in an urban, more socially deprived neighbourhood. The nurses’ role included measurement of weight and blood pressure, patient education on diabetes, including foot care, foot screening, and a referral for 3 or 6-montly HbA1c test. Doctors, as general practitioners, saw patients for medication review and adjustment, which included treatment for hypertension and cholesterol, and to a lesser extent also patient education. The podiatrists saw diabetes patients with and without known foot problems to conduct foot sensation testing, nail care, to advise on footwear, as well as to provide treatment for calluses, and provide after-care post-amputation; severe trauma such as nail punctures were referred for surgical treatment to the local hospital. In addition, one private general practitioner, with an interest in diabetes, was also interviewed. Participants were recruited purposefully at these health facilities according to a range of experiences with foot disease (from no known diabetic foot disease to amputations), their role as health professionals, and gender (six men in the final sample). The doctors and podiatrists working at the two polyclinics in either the diabetes clinics (held once a week in each polyclinic) or wound dressing clinics were interviewed plus all available nurses. Patient participants were attendees of these specialist clinics. They were identified by healthcare professionals and then approached by the researcher to provide them with study information and ensure their informed consent. In selecting the sample for this study, our aim was to purposefully gain access to a range of experiences and social and clinical contexts in a diverse, information-rich sample.

### Interview schedule and data collection

One investigator (CG), an experienced qualitative non-clinical female researcher, conducted all interviews. The interviews took place in a private room of the polyclinics, plus one interview in the office of the private general practitioner. They lasted 30 to 60 minutes and were recorded after seeking written informed consent from all participants. Interview participants were asked to describe how diabetic foot care was experienced and practised, the particular priority given to foot care, and knowledge and attitudes relevant to diabetic foot care. Health professionals were also asked about the new Step-by-Step programme and how they negotiated issues of institutional priority setting and available resources.

### Data analysis

CG conducted the thematic analysis. The interview transcripts (verbatim transcribed) were openly coded, then indexed and synthesised, theoretically guided through a lens of how competing demands and priorities influence the choices made, by health care professionals and patients, around the delivery and adherence to diabetic foot care. Barriers to diabetic foot care were identified, and where possible complementary facilitators were also identified. Codes, categories and emerging themes were discussed with the second author NU in an iterative, discursive process. To ensure rigor, findings were also refined after presentation to a local professional clinical audience. The data analysis process was aided by the open access data management software Weft QDA.

### Ethics

The study was approved by the Institutional Review Board of the University of the West Indies and the Barbados Ministry of Health – and approval for recruitment in polyclinics was granted by the Barbados Chief Medical Officer. Taking the small setting of this study into consideration, no detailed characteristics of the participants are reported, and original quotes are presented with generic labels of ‘patient’ (P) or ‘health professional’ (H) to ensure that no identifiable information is revealed. This was in particular important because there were a very limited number of doctors and podiatrists present at each clinic who were all interviewed; labelling them would directly identify them. However, we have numbered the labels in order to show whether quotes come from the same or different individuals.

## Results

The analysis identified three broad themes: the priority given to glycaemic control, tending to have the effect of relegating other aspects of diabetes care to lower priority; the resistance to new professional roles concerning foot care, particularly within the context of limited resources; and a reliance on the ability of patients not only to manage their diabetes on a day to day basis, but also to negotiate access to diabetic foot care. Under each of these three themes we group findings into barriers and potential facilitators to diabetic foot care.

### Priority of glycaemic control

#### Barriers

Health professionals reported that central to their clinical diabetes management is to achieve diabetes (glycaemic) control in their patients. Foot care was described as a secondary treatment aim following tight glycaemic control. “*[W]e aim for optimum control of blood glucose, we try to get the HbA1c under 7.*” (Health professional, H10) “*The foot care and the diet and the exercise will follow. . . So yes, blood sugar control is first, the other aspects of care come after.*” (H1) Health professionals reported poor diabetes control in many patients, often attributed to poor compliance, and a large percentage of patients with complications. General resource constraints were also reported to require this emphasis on glycaemic control. *“We are very short [of staff]. And the time you would like to spend with each client, sometimes it’s not adequate.*” (H3).

In patients, clinical diabetes control targets also translated into a focus on numbers. “*I do the H1, H1N1 [sic; HbA1c] test, that is de overall sugar level test. So the more you eat more sugar that test goes and goes. And if you go over a certain number, I think it’s over 9, den dey put you now on a higher medication. . . . So I try to cut back on de sweets to keep my sugar levels down.”* (Patient, P2) Patients reported a personal priority setting on fulfilling certain targets set by their health providers. Diet and exercise were reported by most patients as their main lifestyle change or self-care priority to achieve the lower HbA1c levels. Foot care did not necessarily fit this objective of keeping blood sugars down. Health professionals also worried that many patients do not focus on any type of diabetes self-management. “*I don’t really think that patients understand the complications that come with diabetes . . . [T]hey take the medication if they want and taking care of the feet they don't really see it as necessary.*” (H9).

On a side note, for some patients, socio-economic and personal challenges clearly took priority over their overall diabetes self-management. Patients did not explicitly mention this but health professionals were keen to highlight that their patients lead very challenging lives. *“[F]or some, there’re just so much other challenges they perceive that are a lot bigger than the diabetes, so the management takes a side line while they’re tryin’ to focus on their family or stress issues they have.*” (H3) “*Yeah, other issues get in their way, the struggle with poverty, finding a job, the expense of the better foods*.”(H10).

#### Potential facilitators

A focus on diabetes control, however, does not mean an entire disregard of other diabetes management such as foot care, or at least attempts to manage constrained resources efficiently: “*[O]ne of the advantages of keeping your A1c down below 6 is that, as it is they got three of four monthly appointments and they have to come, and they sit and they wait, and have everythin’ done, their foot screened, whatever.*” (H4) Many health professionals also reported that the implementation of a foot care programme led to increasing awareness for foot screening in staff: “*Yeah I think the Step by Step [programme] is reaching all the professionals because the doctors here they are involved in doing the foot inspection and foot examination and reporting of the findings.*” (H5).

Patients’ focus on glucose control and numbers could also be adapted in helping them to understand the stages of diabetic foot disease and ‘at risk feet’ categories. Participants suggested that if health professionals would adopt the – very simple – numbering system of risk category 0 (no loss of protective sensation, no peripheral arterial disease, no deformities) to risk category 3 (history of ulcer or amputation [15]) as widely as HbA1c, this could guide patients’ foot care practices and attention. *“Yes, [patients need to know] ‘I’m a 3 . . . I need to check my feet every day.’. . . [S]ame as havin’ the numbers of HbA1c. So yes, they need numbers so they can fix their level of care. ‘I’m at a 0 so I try hard not to get to 2 or 3.’ You need to know your risk category.”* (H1).

### Resistance to changing professional roles

#### Barriers

As another category of barrier to diabetic foot care, health professionals reported the difficulty to challenge established systems, roles and expectations. In particular in regard to the new Step-by-Step foot care programme, nurses felt that their work duties and the limited materials and time available did not allow for this extended role. “*[F]rom my point of view you’re better able to examine the foot . . . But in terms of the actual [care…] we don’t have the [time], we can’t do any of the actual foot care just the examination.”* (H2) In their view, these time constraints only allowed for screening and referrals rather than care. *“So you find you need to refer to podiatry. . . . So then you kind of increasin’ [the podiatrist’s] workload.*” (H2) Underlying this seems to be a clear description of professional roles and their duties, or at least an institutional culture to adhere to these roles. “*[In] some clinics doctors don’t do the screenings, because they don’t think it is their part to do it, and some nurses would think ‘Well I am not the one who was trained to do the screening . . .’.*” (H6) While interviewed health professionals had the impression that screening and referral seem to have increased recently, some felt that this was not very different to their original clinical practice: “*‘Cause what we were doin’ before was checkin’ the feet and if you find anything that was wrong, then you can refer to podiatry or to the doctor to have the foot look at.*” (H2).

Patients also resisted transformed professional roles, and firmly ascribed care and treatment scope to nurses, doctors and podiatrist, with highest priority and trust placed with doctors. “*But sometimes patients tend to think well if the doctor says ‘this’, that is what they will do. The podiatrist is only . . . here to cut nails.*” (H6).

Although the doctor patient relationship in Barbados is typically described as ‘paternalistic’, this study found that patients pursued some consumerism in their choice of out-of-pocket private care. Patients tended to show a preference for private over public care, with the inherent assumption that private care offers better quality. “*I go to the private doctor because I don’t want to be hospitalised.*” (P5) “*I do have some patients who say would go to the polyclinics twice a year but yet then, they’re kind of saving up their money so they could come to [private practice] for, as they put it, a proper check because they perceive in the polyclinic the doctors just rewrite prescriptions.*” (H11) However, financial barriers in the current economic climate were reported to change the preference of some patients for private care to public care instead. “*I finished with the private doctors. ‘Cause I realised they were takin’ away my money and tellin’ me that my feet is getting’ better and then I end up in the hospital.*” (P5) “*[W]e are noticing that with the downturn in the economy that a lot of persons who would previously receive their podiatry treatment outside [the public system] they are returning to the [poly]clinics.*” (H1) However, in the resource-constrained public health system, even opting for public care requires a certain level of private expenses, such as for materials and foot wear, and patients complained about these out of pocket expenses in the public system. “*I have two pairs of them [diabetic foot wear] and they are expensive. I bought them 350 Dollars [Barbadian; equivalent to USD 175] for a pair. You understand? So 700 Dollars for these two pairs of shoes. But one of the pairs is a dress shoe*.” (P6).

#### Potential facilitators

Although barriers posed by changing professional roles were identified, the new Step-by-Step programme was also described as a facilitator, in particular by nurses. Increased referral and informed screening was also seen as a positive development in diabetic foot care: “*But at least we can spot things, you know, and get them referred quickly.*” (H2) Although many nurses felt that the programme did not train them well enough for delivering care beyond screening, their concerns also showed that there was a general willingness to acquire new skills and extend their professional roles. “*Yeah, I thought it was helpful in the beginning. But I thought it needed to go another step. ‘Cause I thought we needed to do a little bit more of debriding, that sort of thing. [It was] Basic, right?*” (H4).

The willingness of many patients to pay for private care might be seen as a potential facilitator to improved foot care, particularly if this could be translated to improved care within the public system. A health professional suggested: “*Again though cost is a factor, in term of trying, for the wound management part, trying to get them [the patients] to contribute, I think one of the biggest problems in this country is we have not been socialised to paying for our healthcare, it’s been given to us for so long.*” (H5) While patients complained about extra out-of-pocket costs in the public sector, these were largely accepted. “*It’s only certain medication that we have to buy. Like foam, foam dressin’ for my foot.*” (P6) Some of these patients also saw this as an opportunity to customise their care. “*There’s only certain things that you get at the public clinics. The other things that you’d think would be better for you that they don’t have, then you have to buy it yourself. . . . So that’s what I do. I buy what I want and I walk with it.*” (P6).

### Reliance on ‘self-care’ ability

#### Barriers

Finally, health professionals emphasised the important of ‘self-care’ as an essential focus in successful diabetes care. “*I belief self-management of diabetes is the key role. I tell those patients to come here three, four times a year on average and the rest, the 360 days is up to them how they manage their diabetes.*” (H3) However, ‘self-care’ was seen as important not only for the day to day management of diabetes but also in being able to access scarce health care resources. In regard to foot care, for example, the limited availability of podiatry appointments required a great deal of active engagement from the patients. “*Sometimes ‘is difficult [to get a podiatry appointment …]. Like today I went to make an appointment and they say they don’t have any dates. . . . So I have to put that in my mind ‘well, let me call [at a later date] and see if there are any dates available*. . . . *[T]hey will send in dates for certain period. And people will call in and make appointments. And when those are full you have to wait for the podiatrist to give you another set of dates. […] But it means I have to remember to call back.*” (P4) Those patients who were successful in their diabetes self-management and had reached a certain level of self-efficacy and self-reliance were also the ones who accessed care provision most confidently. Less active patients who may be in greater need of treatment appointments were more likely to fail securing scarce appointments.

#### Potential facilitators

Patient education sessions are run in all public care facilities and aim to increase self-management in diabetes patients. For example, lectures delivered in the seating area of the polyclinic while patients wait for appointments, address a range of topics such as ‘eating right during Mango season’. While these lectures are delivered by nurses, the interviews revealed that patients who identified themselves as active self-carers often informally acted as peer educators. “*Sometimes, when I come to the clinic, like, a lady was dere sittin’ and talk and she was dere tellin’ me that every now and den her sugar level always high and she can’t seems to get it controlled. . . . And den she was tellin’ me about the fruits. When you eat fruits you got to drink enough water. Because the fruits contain its natural sugars. So I was tellin’ her . .* .” (P2) There were also reports by interviewees that active self-managing patients can make a positive impact on health professionals’ attitudes and practices and facilitate change in foot care practice. “*Yeah, I think [there is] a greater awareness of the feet because . . . I say to them ‘don’t leave the room, remind the nurse that your feet haven’t been looked at.’ […A]nd a lot of the patients . . . when they come they’re saying to me, ‘nurse I’m supposed to have my feet done’*.” (H5) “*Yes. . . . I wear the stockings. I don’t have them on because I come to clinic, I want nurse and want doc to see the feet.”* (P3).

## Discussion

This qualitative exploratory study aimed to investigate barriers to diabetic foot care from the perspectives of health care providers and patients, with a particular focus on how foot care is approached given other priorities and constrained resources. Three broad types of barrier were identified. The first is the priority given to glycaemic control, with the effect of pushing foot care to a lower level of concern for both health carers and patients. The second barrier was identified specifically by health carers, and that is resistance to taking on new care roles. Finally, patients reported that they often needed to be proactive and self-reliant to access health care, particular podiatry appointments.

The study has several strengths. It was undertaken in a setting that is typical of many middle to high income developing countries, with a high prevalence of diabetes and a health care system that is challenged by this. The participants in this study came from a broad range of health carers and people with diabetes, providing insights into their perspectives. Previous qualitative studies concerning the diabetic foot include those that have examined patient knowledge about foot complications [8], the distressing and socially isolating experiences of foot complications [9], and the perceived lack of self-control in avoiding further ulceration [10]. These studies were undertaken in developed country settings and ours therefore complements them both in terms of its setting and in the focus on barriers to care. Our study, of course, also has limitations. The study was small, particularly when subgroups, such as patients (*n* = 9) and health carers (*n* = 11), are considered separately. The size of the study was in keeping with its exploratory nature, being intended to inform further investigation. In addition our study was largely limited to the public sector and interviews with larger numbers of different categories of health carers and patients may have provided additional insights. It might also be the case that patients with particular characteristics (e.g. those with a history of ulceration) might have reported different types of barriers compared to those who have not experienced foot problems. The numbers of participants with different foot problems, while covering the full range from normal feet to a history of major amputation, was too small to explore this in greater depth. Participants had been selected by the health carers to provide this range – which is a potential limitation, as we were not able to select more neutrally from, for example, medical records.

With the exception of the specific issue of accessing podiatry, the three broad categories of barrier that patients and health carers identified could also apply to other aspects of diabetes care. First, an emphasis on tight glycaemic control was reported to dominate the thinking of both health carers and patients, pushing into second place other aspects of diabetes care, including foot care. In fact, only one interviewee spoke about targets for blood pressure and lipid control, although of course we must emphasise that our study population was small and not intended to be representative in a quantitative sense. Nonetheless, this finding resonates with recent concerns expressed in the literature on reducing cardiovascular risk in people with diabetes. It has been argued that there is an overemphasis on costly tight glycaemic control (i.e. aiming for an HBA1c of 7 % or less) to the potential detriment of highly cost effective approaches to blood pressure and cholesterol control [16, 17]. In addition, randomised controlled trial evidence suggests that tight glycaemic control may have no benefit on cardiovascular risk, or may even in some patients increase risk [18]. In contrast, a study that estimated the cost effectiveness of diabetes interventions in developing countries highlighted three as being potentially cost saving: moderate glycaemic control (HbA1c <9 %), blood pressure control and foot care in people at high risk of ulceration [6]. Estimates of cost effectiveness in a developed country setting, the United States, also indicate that comprehensive foot care is potentially cost saving [5].

A second barrier identified was resistance to changing professional roles, with nurses in particular being expected to take more responsibility for foot care. Studies in health services outside the field of diabetes have noted how professional roles are increasingly changing as part of healthcare restructuring (as in this study with the introduction of the Step-by-Step foot care programme) and that resistance is a common response to this. Resistance is linked to shifts in responsibilities, work remit and load, and decision-making power [19]. In particular, there is a growing body research on ‘task shifting’, the redistribution of tasks within a healthcare team in settings of human resource shortage [20, 21]. Barriers that mirror concerns voiced in this study, identified in largely African settings, include lack of confidence in staff because of inadequate training [22] and unavailability of materials to adequately perform new work roles [23]. Yet task shifting is commonly seen as an essential part of the health care response in developing countries to the rising burden of non-communicable diseases [24]. Studies from higher income settings (mainly Sweden and UK) also highlight that sustainable task shifting is affected by lack of coordination and on-going training, and that task shifting creates uncertainties in inter-professional relationships and ‘grey areas’ of work duties within nonetheless hierarchical clinical structures [25].

Finally, our findings highlighted the inescapable barrier of limited access to podiatry services, a situation reported to be common to the vast majority of developing countries [26]. Barbados has roughly one podiatrist in the public health system for every 10,000 people with diabetes. Although government clinics are geographically accessible and care is free at the point of use, patients needed nonetheless to be proactive in negotiating the appointments system, thus tending to favour access for patients with the time, motivation and skills to do this.

## Conclusion

In conclusion, this qualitative exploratory study in a setting known to have a very high of incidence of diabetes related amputations found three broad categories of potential barriers to effective foot care. These can summarised as barriers related to: goal setting for diabetes care (potential over emphasis on tight glycaemic control); potential resistance to task shifting within the context of scarce human resources; and barriers in access to care posed by overburdened appointment and referral systems to podiatry that can mean that only proactive patients access this specialist care. Potential facilitators were identified against these three categories and included the willingness of health carers and patients to use numbers (as for HbA1c) in describing diabetes control, and this might be used in a person with diabetes knowing their ‘foot score’. Although there was evidence of resistance from health carers to new roles, there was an expressed willingness to take on new roles so long as clearly defined and supported, emphasising the need for training and clearly defining and respecting roles amongst all those involved in diabetic foot care. Finally, there is the potential to make use of patients who are proactive and knowledgeable at negotiating the health care system as educators and guides for other patients. The barriers and potential facilitators identified in this study are worthy of further investigation in order to assist in the design of interventions to reduce the burden of diabetic foot in this and similar settings.
